# Paraoxonase 1 Enzyme Activity in Patients With Isolated Coronary Artery Ectasia

**DOI:** 10.7759/cureus.83703

**Published:** 2025-05-08

**Authors:** Vedat Aslan, Sait Terzi, Kemal Yeşilcimen

**Affiliations:** 1 Department of Cardiology, School of Medicine, Istinye University, Istanbul, TUR; 2 Department of Cardiology, Dr. Siyami Ersek Chest Cardiovascular Surgery Training and Research Hospital, Istanbul, TUR

**Keywords:** coronary artery angiography, coronary artery anomaly, coronary artery disease, coronary artery ectasia, paraoxonase 1

## Abstract

Objective: Paraoxonase 1 (PON1) is an organophosphate ester hydrolase associated with high-density lipoprotein (HDL). The recently emphasized function of PON1 activity is its antiatherogenic activity. PON1 is always found together with HDL in plasma, and PON1 is responsible for the protective effect of HDL against low-density lipoprotein (LDL) oxidation. Considering this role in cardiovascular diseases and its antioxidant properties, PON1 activity may be a valuable biomarker in predicting coronary artery ectasia (CAE), whose pathogenesis has not yet been fully elucidated. The aim was to reveal the relationship between PON1 activity and isolated CAE.

Method: This prospective case-control study's population comprises 5240 patients who underwent coronary angiography between December 2009 and April 30, 2010. Thirty patients with isolated CAE (Group CAE) and 25 volunteers with normal coronary arteries (Group Control) who met the inclusion criteria were included in the study. The sociodemographic, clinical, and anatomical characteristics and lipid profiles of the patients were analyzed. Groups CAE and control were compared in terms of PON1 activity levels.

Results: There was no significant difference between Group CAE (n=30) and Group Control (n=25) in terms of sociodemographic and clinical characteristics (p>0.05). There was ectasia in a single coronary artery in 19 patients (63.3%). The right coronary artery (RCA) was ectasian in 17 patients (38.6%) and was the most affected vessel. According to the Markis classification, the most common type of ectasia was type IV ectasia, which was seen in 14 patients (46.7%). When lipid profiles were compared, no difference was observed between the groups. PON1 activity levels were significantly lower in the CAE group than in the control group (Group CAE=127.5 U/L; Group Control=177.0 U/L; p<0.001).

Conclusions: This study's findings suggest a significant correlation between low PON1 activity levels and the development of isolated CAE, suggesting that PON1 activity may play a potential role in the pathophysiology of CAE. Based on the association demonstrated between isolated CAE and PON1 activity in our study, future research may investigate the potential use of PON1 as a biomarker.

## Introduction

Coronary artery ectasia (CAE) is characterized by the dilation of a coronary artery to 1.5 times or more than the diameter of the adjacent normal segment. It is associated with slow coronary flow, endothelial injury, and thrombus formation. CAE may involve multiple segments of the coronary arteries and can present as either localized or diffuse disease [1,2]. Although often asymptomatic, CAE can also present clinically with stable angina pectoris (AP), unstable AP, acute myocardial infarction (MI), or symptoms of heart failure [3]. When the vascular wall was examined histopathologically in CAE, it was found that diffuse degeneration in the intima and media layers, smooth muscles were replaced by hyalinized collagen, and the vascular wall was thinned [4]. CAE is commonly associated with coronary artery disease (CAD), but it may also occur in isolation without significant coronary stenosis [1,2]. The coexistence of CAE and CAD suggests a potential overlap in their inflammatory pathogenesis [2-4]. Patients with CAE generally exhibit lower levels of high-density lipoprotein cholesterol (HDL-C) and higher levels of low-density lipoprotein cholesterol (LDL-C) [4]. Despite this association, the underlying mechanisms of isolated CAE remain unclear and controversial.

Oxidative stress and chronic inflammation are believed to play a role in the disruption of arterial wall integrity and the development of vascular dilatation [5]. The paraoxonase (PON) gene family consists of three members - PON1, PON2, and PON3 - located on the long arm of chromosome 7 (7q21-22) [6]. Among these, PON1 is the most extensively studied and is closely associated with HDL particles [7].

The anti-atherogenic function of PON1 has received particular attention in recent years. This enzyme, which circulates bound to HDL, detoxifies peroxidized lipids and prevents the accumulation of lipid peroxides in both HDL and LDL particles, thereby reducing oxidative stress. In addition, it supports endothelial function and may modulate vascular inflammation through its anti-inflammatory properties [8]. Notably, the protective effect of PON1 against LDL oxidation has been reported to be stronger than that of vitamins A and E [9].

Reduced PON1 activity is associated with enhanced oxidative and inflammatory processes that may contribute to the development of CAE. However, mechanistic evidence supporting this association remains limited [10,11]. Indeed, previous studies have demonstrated significant correlations between low serum PON1 levels and several forms of CAD, including acute MI [8,9,12].

Only one study to date has directly investigated the relationship between PON1 activity and CAE. In that study, both HDL and PON1 activity levels were significantly lower in patients with isolated CAE compared to healthy controls, suggesting that reduced PON1 levels may be closely linked to the pathogenesis of the disease [13]. Nevertheless, the limited number of studies in this area highlights the need for further investigation.

## Materials and methods

Study design

This study is prospective and designed as a case-control study. The local ethics committee approved the study protocol (06.07.2009, 4869). The study was conducted on the ethical issues stated in the Declaration of Helsinki. Informed consent was obtained from the patients and healthy volunteers.

The study population comprises 5,420 consecutive patients who underwent coronary angiography at the Cardiology Clinic of Prof. Dr. Siyami Ersek Chest Cardiovascular Surgery Training and Research Hospital between December 1, 2009, and April 30, 2010. Angiography was performed using standard 6F Judkins right and left catheters via femoral artery puncture and without the use of nitroglycerin. It was viewed in at least four projections for the left coronary system and at least two projections for the right coronary system and recorded in digital memory. Coronary angiograms were reviewed by three experienced interventional cardiologists who were unaware of the patient's clinical characteristics and comorbid diseases. In cases where no consensus was reached, the acceptance or exclusion of the ectasia diagnosis was resolved by the consensus of two (2/3) of the three cardiologists. Ectasia and CAD diagnoses were made using quantitative measurement methods of the angiography device. Additionally, Fleiss’ kappa coefficient was calculated to assess inter-rater reliability. The obtained value of 0.92 indicates a very high level of agreement among the observers.

Coronary artery ectasia, defined as a localized or diffuse dilatation of at least one coronary artery by 1.5 times or more than the diameter of the adjacent normal segment, was observed in 1.9% of all patients (102 patients). The absence of critical stenosis (more than 50% occlusion in any of the coronary arteries) in CAE patients was defined as “isolated CAE” [1-3,10]. Isolated CAE was diagnosed in 0.95% of all patients (52 patients). Conditions that could affect PON1 activity and diseases that progress with CAE were included as exclusion criteria in our study [11-13] (Table 1).

**Table 1:** Inclusion and exclusion criteria Sources: Refs. [11-13]

Inclusion and Exclusion Criteria
Inclusion criteria
Patients over 18 years of age
Localized or diffuse dilatation of at least one coronary artery by 1.5 times or more than the diameter of the adjacent normal segment
Exclusion criteria
Critical (more than 50% stenosis in any vessel) coronary artery stenosis
Moderate or severe heart valve disease
Cardiomyopathy
Endocrine(hypo- and hyperthyroidism)/metabolic disorders(Marfan syndrome, Ehlers-Danlos)
Statin usage
History of Kawasaki disease
Connective tissue disease
Steroid or nonsteroidal drug usage
Active or a history of malignancy
Patients with familial hypercholesterolemia

**Figure 1:**
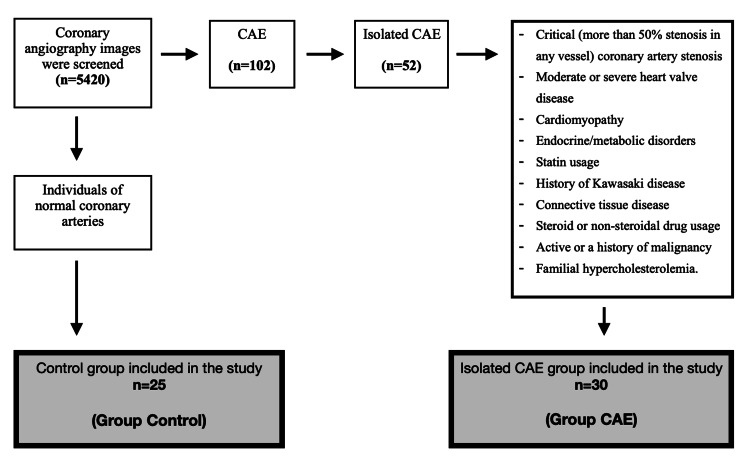
Study flow diagram CAE: Coronary artery ectasia

Twenty-two patients were not included in our study due to the exclusion criteria, and 30 isolated CAE patients were included in the study. The control group consisted of volunteers who did not have CAE or coronary artery disease and agreed to participate in the study (Figure 1).

PON1 measurements

No medications were administered to the patients from the time of hospital admission until the completion of coronary angiography. Blood samples were collected into biochemistry tubes by the primary investigator physician within a maximum of 30 minutes following the completion of the coronary angiography procedure in each case. During this period, all patients were kept at rest. Patients who did not meet these criteria were excluded from the study under the designation of “inappropriate data.” Immediately after blood collection, the samples were centrifuged at 4,000 rpm for 10 minutes to separate the serum. Hemolyzed serum samples were excluded from the study. The serum samples were stored at −20°C until the day of analysis. PON1 activity measurements were performed in the laboratory using the same equipment and technique previously employed in studies on the PON1 enzyme, based on the method originally developed by Furlong and modified by an experienced biochemist. All measurements were conducted in a double-blind manner by the same technician. According to the manufacturer’s data, the intra-assay and inter-assay coefficients of variation (%CV) were 5.2% and 7.8%, respectively [14]. For the reaction mixture, 1.32 mM CaCl₂ and 2.63 M NaCl were added to 0.132 M Tris-HCl buffer (pH 8.0). For each assay, 300 µL of 5 mM paraoxon (O,O-diethyl-O-p-nitrophenyl phosphate; Sigma Co., London, UK) was freshly prepared and added to the mixture to inhibit potential butyrylcholinesterases. The analysis was initiated by adding 60 µL of serum to the reaction mixture. To determine PON1 activity, the increase in absorbance caused by the enzymatic hydrolysis of paraoxon, resulting in the formation of p-nitrophenol, was measured spectrophotometrically at 405 nm and 37°C at 1-minute intervals for five minutes using a Biosystem BTS310 spectrophotometer (Barcelona, Spain). The average change in absorbance per minute (∆A) was calculated from these readings, and paraoxonase activity was determined using the molar extinction coefficient of 18.05 × 10³ M⁻¹ cm⁻¹. One unit of PON1 activity was defined as the amount of enzyme that generates 1 µmol of p-nitrophenol per minute. The results of paraoxonase activity were expressed in units per liter (U/L).

Data collection

Sociodemographic and clinical characteristics, laboratory findings, and angiography images of the patients included in the study were recorded from medical files in the interventional Cardiology Unit. The researchers involved in the collection of clinical and laboratory data were blinded to case/control status. Three interventional cardiologists noted angiography features and localization of the CAE patients. The severity of CAE was classified according to the Markis classification system, which is widely used and accepted in previous studies on CAE. The Markis classification is divided into four groups according to the ectatic involvement of the coronary arteries [3,15] (Table 2).

**Table 2:** Markis classification Sources: Refs. [3,15]

Type	Markis classification
Type I	Diffuse ectasia in two or three vessels
Type II	Diffuse ectasia in one vessel and localized ectasia in the other vessel
Type III	Diffuse ectasia in only one vessel
Type IV	Localized or segmental ectasia

Statistical analysis

All statistical analyses were performed using Statistical Product and Service Solutions (SPSS, version 13.0; SPSS Inc., Chicago, IL). Sample size and power calculations were conducted using the PASS software package (version 11.0; NCSS LLC, Kaysville, UT) to ensure a study power of 80% with a two-tailed alpha level of 0.05. Continuous variables are presented as mean ± standard deviation, and categorical variables as frequencies and percentages. The normality of distribution was assessed separately for each group using the Shapiro-Wilk, Kolmogorov-Smirnov, and Anderson-Darling tests. Between-group comparisons were conducted using the independent samples T-test for normally distributed variables and the Mann-Whitney U test for non-normally distributed variables, considering potential group size differences. In cases where more than two groups were compared, the Kruskal-Wallis test was used to assess differences in non-normally distributed continuous variables. Categorical variables were analyzed using Pearson’s chi-square test or Fisher’s exact test, where appropriate. Bonferroni correction was applied to adjust for multiple comparisons, particularly in baseline characteristics. Correlation analysis between serum PON1 levels and variables such as age and lipid profile was performed using Spearman’s rho due to non-normal distributions. These analyses were exploratory and based on prior literature. In addition, multivariate linear regression analysis was performed to determine independent predictors of serum PON1 levels. Variables that showed significance in univariate analyses or were clinically relevant (e.g., age, BMI, smoking, lipid parameters) were included in the regression model. Assumptions of linearity, independence, homoscedasticity, and multicollinearity were checked prior to analysis.

## Results

Among all patients who underwent coronary angiography, isolated CAE was identified in 52 individuals (0.95%). Of these, 30 patients diagnosed with isolated CAE were included in the study. The mean age of the CAE group was 56.9 ± 10.7 years, and 63% (n=19) were male. Table 3 presents the comparison of sociodemographic and clinical characteristics between the CAE group (n=30) and the control group with normal coronary anatomy (n=25). There were no statistically significant differences between the two groups in terms of age, sex, smoking and alcohol use, hypertension, or diabetes (p>0.05).

**Table 3:** Sociodemographic and clinical characteristics of the study groups The table presents data as the mean ± standard deviation (denoted by '†') and number of cases with percentages (denoted by ‘‡’). The statistical significance between groups is evaluated using the independent samples T-test ('**') and Pearson chi-square or Fisher's exact test ('*'). BMI: Body mass index, CAE: Coronary artery ectasia, ES (φ): Effect size, dF: Degrees of freedom

Variables	Group CAE (n=30)	Group Control (n=25)	df	ES (φ)	p-value
Age (year)^ †^	56.9 ± 10.7	54.8 ± 9.4	53	204	0.449**
BMI (kg/m^2^)^ †^	30.4 ± 4.1	29.2 ± 7.4	53	193	0.501**
Sex^ ‡^					
Female	11 (36.7)	11 (44.0)	1	0.075	0.782*
Male	19 (63.3)	14 (56.0)			
Smoking^ ‡^	7 (23.3)	7 (28.0)	1	0.053	0.932*
Alcohol^ ‡^	7 (23.3)	4 (16.0)	1	0.091	0.735*
Comorbidities^ ‡^					
Diabetes mellitus	10 (33.3)	6 (24.0)	1	0.069	0.818*
Hypertension	20 (66.7)	15 (60.0)	1	0.102	0.645*

Similarly, there were no significant differences between the groups in lipid parameters, including LDL-C, HDL-C, and triglyceride levels (p>0.05) (Table 4). After the Bonferroni correction was applied to reduce the risk of type I error in multiple comparisons, the results remained unchanged.

**Table 4:** Laboratory investigations of the study groups Data are expressed as mean ± standard deviation (‘†’). Statistical differences between the two groups are assessed using the independent samples T-test ('*') and the Mann-Whitney U test ('**'). CAE: Coronary artery ectasia, LDL-C: Low-density lipoprotein-cholesterol, HDL-C: High-density lipoprotein-cholesterol, PON1: Paraoxonase 1, ES (φ): Effect size, dF: Degrees of freedom

Variables	Group CAE (n=30)	Group Control (n=25)	df	ES (φ)	p-value
LDL-C (mg/dL)^ †^	115.3 ± 36.5	122.2 ± 36.7	5	-0.188	0.492*
HDL-C (mg/dL)^ †^	44.5 ± 10.85	46.0 ± 8.85	3	-0.095	0.554**
Triglyceride (mg/dL)^ †^	169.1 ± 57.2	170.4 ± 54.8	5	-0.024	0.928*
PON1 (U/L)^ †^	127.5 ± 10.82	177 ± 26.18	3	-0.964	<0.001**

**Figure 2:**
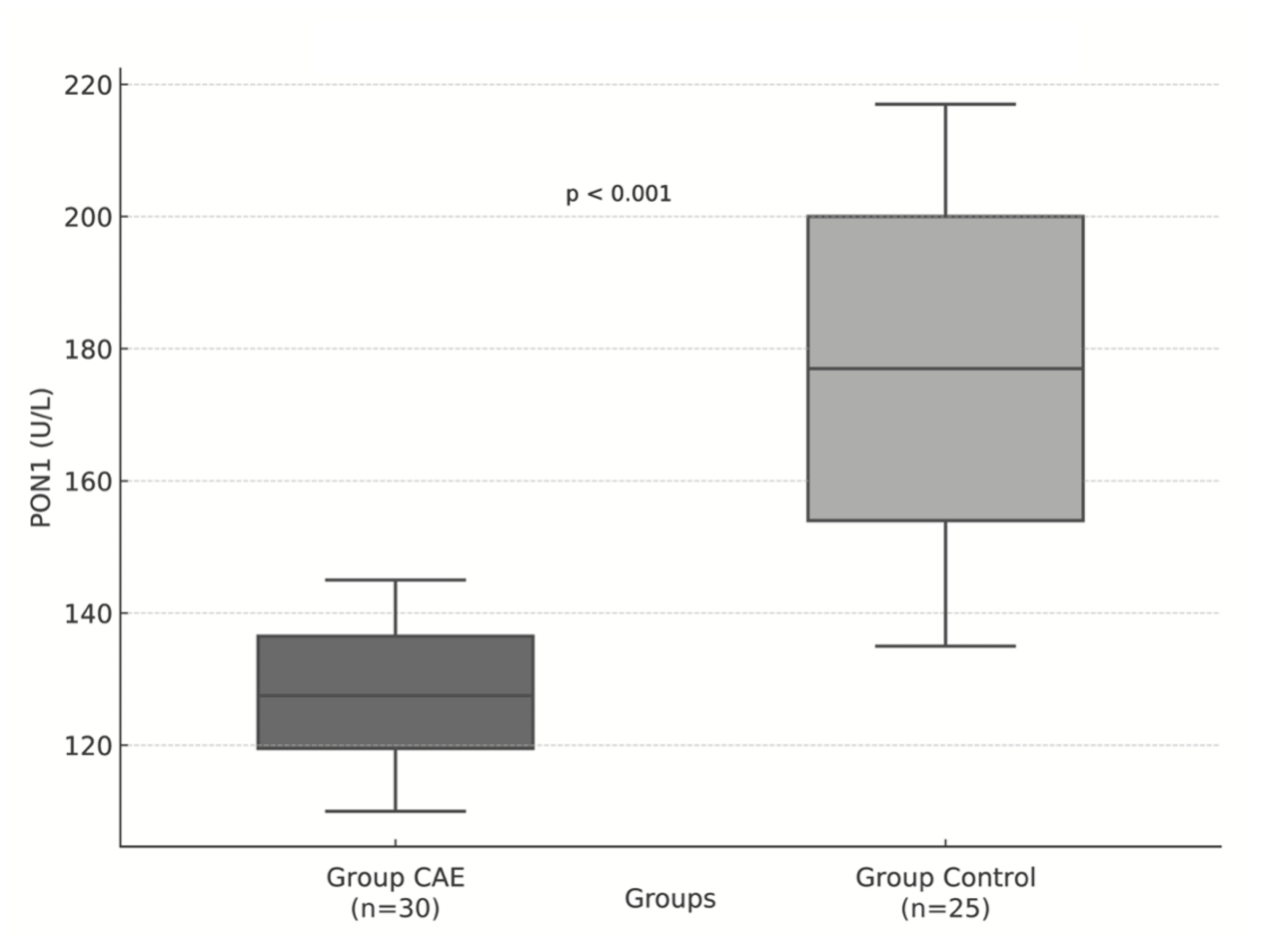
Graphical representation of PON1 activity levels in Groups CAE and Control PON1: Paraoxonase 1, CAE: Coronary artery ectasia

On the other hand, PON1 activity levels were significantly lower in the CAE group compared to controls (127.5 U/L vs. 177.0 U/L; p<0.001). This difference is illustrated graphically in Figure 2.

To evaluate whether the PON1 level was independently associated with CAE, a multivariate logistic regression analysis was conducted. CAE was modeled as the dependent variable, with PON1 activity as the primary independent variable. Age, BMI, LDL, HDL, triglycerides, alcohol use, smoking status, diabetes, and hypertension were included as potential confounders. None of these variables significantly affected the relationship between PON1 and CAE (all p>0.05).

Angiographic evaluation showed that 19 patients (63.3%) had ectasia in a single coronary artery. A total of 44 ectatic vessels were identified in 30 patients. The most frequently affected vessel was the right coronary artery (RCA), observed in 17 patients (38.6%), followed by the left anterior descending artery (LAD) and the circumflex artery (Cx). According to the Markis classification, the most common type of ectasia was type IV, seen in 14 patients (46.7%) (Table 5).

**Table 5:** Features of the ecstatic coronary vessels in Group CAE The data are presented as the number of cases with corresponding percentages (denoted by '‡'). CAE: Coronary artery ectasia

Variables	Group CAE (n=30)
Number of ecstatic vessels^ ‡^	
One	19 (63.3)
Two	8 (26.7)
Three	3 (10.0)
Localization (n=44)^ ‡^	
Right coronary artery	17 (38.6)
Left anterior descending artery	13 (29.5)
Circumflex artery	13 (29.5)
Left main coronary artery	1 (2.3)
Markis classification^ ‡^	
Type I	3 (10.0)
Type II	8 (26.7)
Type III	5 (16.7)
Type IV	14 (46.7)

The relationship between the Markis classification and biochemical and clinical parameters was assessed using the Kruskal-Wallis test. A significant difference was observed between groups in terms of triglyceride levels (p<0.05). Post-hoc Dunnett’s T3 test revealed that this difference was significant between type 2 and type 4 (p=0.007) and between type 3 and type 4 (p=0.010). No significant differences were found between Markis groups regarding other biochemical parameters or PON1 levels (Tables 6-7).

**Table 6:** Kruskal-Wallis test results for age, lipid profile, and PON1 by Markis groups LDL: Low-density lipoprotein, HDL: High-density lipoprotein, PON1: Paraoxonase 1; df: Degrees of freedom

Variables	df	p-value
Age	3	0.198
LDL	3	0.107
HDL	3	0.516
Triglycerides	3	0.02
PON1	3	0.720

**Table 7:** Descriptive statistics for age, lipid profile, triglycerides, and PON1 according to Markis groups LDL: Low-density lipoprotein, HDL: High-density lipoprotein, PON1: Paraoxonase 1

Variable	Markis Group	N	Mean	Std. Deviation	Maximum
Age	1	2	56.50	3.536	59
	2	8	63.00	12.895	76
	3	5	57.80	12.795	71
	4	15	53.33	8.398	68
	Total	30	56.87	10.686	76
LDL	1	2	158.00	25.456	176
	2	8	92.25	36.023	149
	3	5	130.40	23.734	154
	4	15	116.93	35.528	167
	Total	30	115.33	36.546	176
HDL	1	2	57.00	14.142	67
	2	8	47.50	13.836	79
	3	5	43.60	4.669	50
	4	15	44.93	10.278	72
	Total	30	46.20	10.845	79
Triglycerides	1	2	144.50	27.577	164
	2	8	139.75	40.854	225
	3	5	111.00	22.561	138
	4	15	207.33	49.014	272
	Total	30	169.07	57.241	272
PON1	1	2	119.00	11.314	127
	2	8	126.88	13.357	143
	3	5	127.60	12.178	144
	4	15	128.93	9.445	145
	Total	30	127.50	10.824	145

Within the CAE group, PON1 activity levels were compared across subgroups based on sex, smoking and alcohol use, hypertension, diabetes status, number of ectatic vessels, and Markis classification. No statistically significant differences were observed in any of these comparisons (Table 8).

**Table 8:** Comparison of serum PON1 activity levels in Group CAE (n=30), based on sociodemographic and clinical characteristics Data are expressed as mean ± standard deviation (denoted by '§'). The statistical significance of the differences in PON1 levels between different subgroups is assessed using the Mann-Whitney U test (denoted by '*'). PON1: Paraoxonase 1, CAE: Coronary artery ectasia, ES (φ): Effect size, dF: Degrees of freedom

Variables	PON1 (U/L)^ §^	ES (φ)	df	p-values
Female (n=11)	127.36 ± 9.23	-0.014		0.996*
Male (n=19)	127.58 ± 11.89			
Smoker (n=7)	123.86 ± 10.77	0.255		0.327*
Non-smoker (n=23)	128.61 ± 11.01			
Alcohol Present (n=7)	125.29 ± 12.32	0.143		0.590*
Alcohol Absent (n=23)	128.17 ± 10.05			
Hypertensive (n=20)	127.9 ± 12.64	0.035		0.895*
Normotensive (n=10)	127.3 ± 10.14			
Diabetic (n=10)	122.5 ± 8.98	0.415		0.071*
Non-diabetic (n=20)	130.0 ± 10.99			
One ecstatic vessels (n=19)	128.16 ± 9.92	0.007	2	0.909**
Two ecstatic vessels (n=8)	126.88 ± 13.35			
Three ecstatic vessels (n=3)	125.0 ± 13.11			
Markis classification type I (n=3)	119.0 ± 11.31	0.046	3	0.720**
Markis classification type II (n=8)	126.88 ± 13.36			
Markis classification type III (n=5)	127.60 ± 12.18			
Markis classification type IV (n=14)	128.93 ± 9.45			

When the number of ectatic vessels was taken into account, Kruskal-Wallis analysis showed no significant differences in PON1 activity or lipid parameters (LDL, HDL, triglycerides) between groups (p>0.05). These findings suggest that the extent of ectatic involvement may not be directly associated with changes in lipid profile or PON1 activity (Table 9).

**Table 9:** Kruskal-Wallis test results comparing lipid profiles and PON1 levels by the number of affected vessels LDL: Low-density lipoprotein, HDL: High-density lipoprotein, PON1: Paraoxonase 1, df: Degrees of freedom

Variable	Kruskal-Wallis H	df	p-value
LDL	5.976	2	0.050
HDL	4.300	2	0.116
Triglyceride	3.887	2	0.143
PON1	190	2	0.909

Correlation analysis between PON1 activity and age and lipid profile revealed a weak positive correlation only with LDL-C in the CAE group (r=0.234; p<0.05). No significant correlation was observed with other lipid parameters (Table 10).

**Table 10:** Correlation of PON1 activity levels with age and lipid profile in Group CAE The correlation coefficients (r) and their significance (p) are calculated using Spearman's rho correlation coefficient. The correlation coefficients (r) and their significance (p) are calculated using Spearman's rho correlation coefficient. This table is instrumental in understanding how PON1 levels are related to age and various lipid profile parameters in patients with CAE. PON1: Paraoxonase 1, CAE: Coronary artery ectasia, LDL-C: Low-density lipoprotein-cholesterol, HDL-C: High-density lipoprotein-cholesterol

Variables	PON1 (U/L)	
	r	p
Age	-0.091	0.632
LDL-C	0.234	0.214
HDL-C	-0.018	0.925
Triglyceride	-0.067	0.726

To further assess whether the PON1-lipid relationship was specific to CAE, correlation analysis was also performed in the control group. No statistically significant relationships were found between PON1 and LDL, HDL, or triglyceride levels in the control group (p>0.05) (Table 11).

**Table 11:** Spearman's correlation coefficients between PON1 and lipid parameters LDL: Low-density lipoprotein, HDL: High-density lipoprotein, PON1: Paraoxonase 1 enzyme activity

Variable	Spearman's r	p-value	n
Pon1 vs. HDL	0	999	25
Pon1 vs. Triglyceride	-86	684	25
Pon1 vs. LDL	-326	112	25

## Discussion

Although many studies have shown the relationship between low-PON1 activity and obstructive CAD, only a few small-sized studies have been found that show the relationship between isolated CAE and PON1 activity levels. This study investigated the relationship between isolated CAE and PON1 activity. The findings were discussed according to the literature.

According to various studies, the incidence of isolated CAE is between 0.1% and 1.05% and is more common in men [2-4,16,17]. While it predominantly presents as RCA ectasia, it is less common in the circumflex coronary artery (CFx) and LAD ectasia [17,18]. Although publications report all four groups as the most common type according to the Markis classification, type 4 CAE is the most common in large-sized studies [16,19,20]. In our study, the incidence of isolated CAE was determined as 0.95% (n=52), and 63% of these patients were men. Similar to the literature, 38.6% of the patients included in the study were dominated by isolated RCA ectasia. In addition, type 4 CAE was the most common in the patients included in the study.

CAE is associated with many factors, but the pathogenesis and exact mechanism are unknown [21]. The high association with CAD in studies on CAE suggests that there may be common mechanisms in pathogenesis [22-25]. The atherosclerotic process extends from the intima to the media, leading to intimal hyalinization, lipid accumulation, and media disruption due to overexpression of matrix metalloproteinases (MMPs). MMPs cause the degradation of collagen structure by proteolysis of extracellular matrix proteins, resulting in pathological dilatation [26]. Increasing evidence on CAE reveals a strong association of CAE with inflammation (4,22). In addition, Chalikias et al. demonstrated an increased prevalence of positive ANA titers in patients with CAE, indicating an underlying autoimmune disease [27]. A report on the immune-inflammatory response showed significantly higher systemic levels of INF-gamma, TNF-alpha, IL-1ß, and IL-8 and lower levels of IL-2 and IL-4 compared with the control group [2]. Activation of inflammatory cell-mediated matrix-degrading enzymes may weaken arterial wall integrity by causing the vascular wall's breakdown of collagen and elastin fibers, ultimately resulting in an ecstatic appearance of the vascular wall [28-31].

In our study, we observed that PON1 levels were significantly lower in patients with CAE. This finding is consistent with the downregulation of PON1 in an environment where oxidative stress is increased and inflammation is predominant. Low PON1 levels may reduce the capacity of HDL to detoxify lipid peroxides, leading to oxidative damage in endothelial cells. This process may trigger the release of inflammatory cytokines and cause chronic vascular inflammation. Furthermore, decreased PON1 activity may accelerate the oxidation of LDL particles, supporting the accumulation of oxidized LDL and foam cell formation. As a result, extracellular matrix degradation in the arterial wall increases, and the overexpression of MMP-2 and MMP-9 leads to the weakening of elastic tissue. This pathophysiological cascade may be one of the key mechanisms underlying the vascular dilatation observed in CAE.

PON1 is an enzyme involved not only in oxidative stress regulation but also in modulating inflammatory responses. Reduced PON1 activity may enhance the effects of pro-inflammatory cytokines, such as TNF-α and IL-6, thereby exacerbating vascular inflammation and compromising the integrity of the arterial wall, thus facilitating the development of CAE. This interaction suggests that PON1 may play a potential mediating role in both inflammatory processes and vascular remodeling.

The relationship between diabetes and CAE remains unclear. While some meta-analyses suggest that diabetes may prevent CAE by promoting basement membrane thickening and extracellular matrix accumulation [21], others report that diabetes increases the risk of CAE by 19% [32]. In the same study, hypertension was shown to increase the likelihood of CAE by 44% when accompanied by other risk factors, while the adjusted effect of hypertension increased the risk by only 3%. Smoking accelerates the atherosclerotic process, triggers inflammation, and increases ischemia [33]. It also causes constriction in the arterial wall and raises blood pressure. Smoking has been shown to increase the risk of CAE by 53% compared to the control group [32]. However, in our study, no significant association was found between CAE and the presence of DM, HT, or smoking. Although there was a trend toward lower PON1 levels in individuals with diabetes, this difference did not reach statistical significance. This may suggest that PON1 levels could be influenced by diabetes-related oxidative stress.

It is known that inflammation and oxidative stress play an important role in the development of CAD in hyperlipidemic patients [34]. A higher prevalence of CAE has been shown in patients with familial hypercholesterolemia, with higher LDL-C levels, lower HDL-C, and higher LDL/HDL ratios [35]. Similarly, an elevated LDL-C/HDL-C ratio is predictive of the development of CAE [36]. PON1, an antioxidant-active enzyme in HDL-C molecules, was identified in studies conducted by Mackness et al. It effectively prevents inflammatory responses in arterial wall cells by neutralizing biologically active lipids in slightly oxidized LDL and is, therefore, antiatherogenic [37]. Similarly, it may also reduce the development of ectasia with its antioxidant and anti-inflammatory properties. Therefore, it may be expected that PON1 activity will be low in patients with CAE, as in CAD. It has also been reported that low PON1 activity in patients with CAE may increase the amount of oxidized LDL-C and potentially lead to extracellular matrix degradation in atherosclerotic plaques, promoting plaque rupture [38]. The beneficial results obtained in a study with rosuvastatin may be explained by the role of rosuvastatin in suppressing MMP expression and reducing inflammation in CAE patients [39].

Future studies should more comprehensively evaluate whether PON1 could serve as a therapeutic target and assess the effects of statins on this target.

When we examined the relationship between the Markis classification and biochemical parameters, significant differences were observed in triglyceride levels. Kruskal-Wallis analysis revealed statistically significant differences in triglyceride levels between Markis type 2 and type 4 (p=0.007) and between type 3 and type 4 (p=0.010). These findings suggest that different Markis types may influence biochemical parameters. No significant differences were observed between the groups in terms of other biochemical parameters or PON1 levels. This result indicates that, while triglyceride levels may be associated with the Markis classification, other biochemical parameters may not reflect this relationship. Although similar findings have been reported in the literature, the clinical relevance of the association between triglyceride levels and the Markis classification should be clarified through further research.

In the Kruskal-Wallis analysis based on the number of ectatic vessels, no significant differences were found between the groups in terms of PON1 activity or lipid parameters. This suggests that PON1 levels may reflect underlying biological processes such as oxidative stress and impaired antioxidant defense mechanisms, rather than the extent of ectatic involvement. Furthermore, the absence of a relationship between lipid profile and the severity of ectatic involvement supports the view that lipid disorders reflect a systemic metabolic state rather than the degree of vessel involvement. However, the relatively small sample size and possible heterogeneity among the groups warrant cautious interpretation of these findings.

Limitations

Our study has several limitations. First, the relatively small sample size limits the generalizability of the findings. Moreover, the current sample may not have sufficient statistical power to detect moderate but potentially meaningful differences. Therefore, p-values above 0.05 do not entirely rule out the presence of clinically relevant effects. Second, the lack of comprehensive data in the literature on CAE limits the interpretation of PON1 activity within the broader context of cardiovascular diseases. Thus, there is a need for larger prospective studies that include more diverse patient populations and other cardiac pathologies, such as coronary artery disease. Third, patients using statins were excluded from the study due to their potential effect on PON1 levels. While this strengthens internal validity, it may limit the external applicability of the results. Fourth, blood samples were obtained immediately after the invasive procedure. Although this approach offers a practical advantage by enabling timely sample collection, possible hemodynamic and biochemical changes following the procedure may have influenced PON1 levels. Therefore, this should also be considered a limitation. PON1 activity was measured at a single time point. The potential presence of subclinical atherosclerosis and other unmeasured confounding factors in the control group, as well as inter-laboratory variability, should be taken into careful consideration when interpreting the results. Fifth, clinical variables such as the duration, severity, and treatment status of comorbidities such as hypertension and diabetes may influence PON1 levels. However, due to the lack of detailed information on these factors, they could not be accounted for in multivariable analyses. Finally, since isolated CAE is often asymptomatic, determining its true incidence is inherently difficult. Moreover, its low prevalence makes it challenging to reach a statistically adequate sample size, thereby limiting the generalizability of our findings to larger patient populations.

## Conclusions

The findings of this study revealed a significant correlation between low PON1 activity and the development of CAE and suggested the potential role of PON1 activity in the pathophysiology of CAE. Large-scale prospective studies can be conducted to better understand the relationship between PON1 activity and the development of CAE, improve risk stratification, and protect patients from possible major cardiovascular events.
